# Supplementary material for: A UMLS-based spell checker for natural language processing in vaccine safety
Source: BMC Med Inform Decis Mak. 2007 Feb 12;7:3. doi: 10.1186/1472-6947-7-3 (PMC1805499; doi:10.1186/1472-6947-7-3)
Supplement: Additional file 13 — RAP application directory. Sets up directory for RAP (RDF application for PHP) source code [file 1472-6947-7-3-S13.gz › rap/doc/phpdoc/syntax/N3Serializer.html]

xml version="1.0" encoding="iso-8859-1"?


Docs For Class N3Serializer


## Class N3Serializer

Description

Description |
Vars (details)
| Methods (details)

An abstract object.

Root object with some general methods, that should be overloaded.

History:

- 09-10-2002 : First version of this class.

- version: V0.9.1
- author: Chris Bizer <chris@bizer.de>

Located in /syntax/N3Serializer.php (line 45)

```
Object
   |
   --N3Serializer
```

Variable Summary

Description |
Vars (details)
|
Methods (details)

mixed
$anon

mixed
$debug

mixed
$done

mixed
$model

mixed
$prefixes

mixed
$res

mixed
$resourcetext

mixed
$resourcetext\_taken

Method Summary

Description |
Vars (details)
|
Methods (details)

N3Serializer
N3Serializer
()

void
addNSPrefix
(mixed $ns, mixed $prefix, string $s)

boolean
saveAs
(object MemModel &$model, string $filename)

string
&serialize
(mixed &$m, object Model $model)

Variables

Description |
Vars (details)
|
Methods (details)

mixed
$anon
(line 56)

mixed
$debug
(line 47)

mixed
$done
(line 51)

mixed
$model
(line 54)

mixed
$prefixes
(line 49)

mixed
$res
(line 55)

mixed
$resourcetext
(line 52)

mixed
$resourcetext\_taken
(line 53)

Methods

Description |
Vars (details)
Methods (details)

Constructor N3Serializer (line 63)

Constructor

- access: public

N3Serializer

N3Serializer
()

addNSPrefix (line 76)

Adds a new namespace prefix to use.

Unknown namespaces will become ns0, ns1 etc.

- access: public

void

addNSPrefix
(mixed $ns, mixed $prefix, string $s)

- string
  $s

saveAs (line 192)

Serializes a model and saves it into a file.

Returns FALSE if the model couldn't be saved to the file.

- access: public

boolean

saveAs
(object MemModel &$model, string $filename)

- object MemModel
  $model
- string
  $filename

serialize (line 87)

Serializes a model to N3 syntax.

- access: public

string

&serialize
(mixed &$m, object Model $model)

- object Model
  $model

#### Inherited Methods


Inherited From Object

> Object::toString()

Documentation generated on Thu, 7 Jul 2005 13:42:14 +0200 by phpDocumentor 1.3.0RC3
